# Women with polycystic ovary syndrome have poorer work ability and higher disability retirement rate at midlife: a Northern Finland Birth Cohort 1966 study

**DOI:** 10.1530/EJE-22-0027

**Published:** 2022-07-13

**Authors:** Linda Kujanpää, Riikka K Arffman, Eeva Vaaramo, Henna-Riikka Rossi, Jaana Laitinen, Laure Morin-Papunen, Juha Tapanainen, Leena Ala-Mursula, Terhi T Piltonen

**Affiliations:** 1 PEDEGO Research Unit (Research Unit for Pediatrics, Dermatology, Clinical Genetics, Obstetrics and Gynecology), University of Oulu, Oulu, Finland; 2 Medical Research Center Oulu (MRC Oulu), University of Oulu, Oulu, Finland; 3 Department of Obstetrics and Gynecology, Oulu University Hospital, Oulu, Finland; 4 Infrastructure for Population Studies, Faculty of Medicine, University of Oulu, Oulu, Finland; 5 Finnish Institute of Occupational Health, Oulu, Finland; 6 Department of Obstetrics and Gynecology, University of Helsinki, Helsinki University Hospital, Helsinki, Finland; 7 Center for Life Course Health Research, Faculty of Medicine, University of Oulu, Oulu, Finland

## Abstract

**Objective:**

Polycystic ovary syndrome (PCOS) presents with multiple comorbidities potentially affecting function. This was the first general population-based study to evaluate work ability, participation in working life, and disability retirement in middle-aged women with and without PCOS.

**Design:**

This is a cohort study.

**Methods:**

Women with PCOS (*n* = 280) and women without PCOS symptoms or diagnosis (*n* = 1573) were identified in the Northern Finland Birth Cohort in 1966 and were evaluated for self-rated work ability and potential confounders at age 46. Next, incidence rate ratios (IRRs) for disability and unemployment days were extracted from national registers during a prospective 2-year follow-up. Lastly, we assessed hazard ratios (HRs) for disability retirement between 16 and 52 years of age from national registers.

**Results:**

The women with PCOS reported poorer ability to work at age 46, especially due to poorer health. During the 2-year follow-up period, the affected women gained on average an additional month of disability and unemployment days, corresponding to an approximately 25% higher risk for both disability (IRR (95% CI): 1.25 (1.22–1.27)) and unemployment days (IRR (95% CI): 1.26 (1.23–1.28)) in models adjusted for health and socioeconomic factors. Lastly, we found a two-fold higher cumulative risk for disability retirement by age 52 compared to non-PCOS women (HR (95% CI): 1.98 (1.40–2.80)), which remained after adjusting for confounding factors (aHR (95% CI): 1.55 (1.01–2.38)).

**Conclusions:**

PCOS is associated with lower participation in working life already in midlife. Acknowledging PCOS-related multimorbidity, concerted efforts are needed to support sustainable careers for women with PCOS.

## Introduction

Polycystic ovary syndrome (PCOS) is the most common endocrinopathy in women and is increasingly understood to present with numerous comorbidities (1, 2, 3, 4). Indeed, PCOS is not solely a reproductive disorder. The PCOS-related health risks and comorbidities include obesity and a wide range of metabolic, psychological, and musculoskeletal disorders (5, 6, 7, 8, 9, 10). Since such disorders affect daily functioning and are known risk factors for poor work ability and early retirement (11, 12), studies focusing on the effects of PCOS on working life are urgently needed.

We are aware of only one study that has addressed this issue – albeit indirectly – by evaluating the association between PCOS and occupation-based socioeconomic status (SES) (13). This large, cross-sectional Danish study found that women with PCOS (aged 25–60) were more often unemployed or on welfare and had retired early than non-PCOS women. Both lower SES and higher BMI were associated with adverse working life outcomes. However, as this study was hospital-based, it likely included more severe cases of PCOS. To date, longitudinal, general population-based evidence on work ability in relation to PCOS is lacking.

Here, we examine at the level of general population the consequences of PCOS on work ability and disability until midlife, considering several potential confounders. By linking rich survey data from the Northern Finland Birth Cohort 1966 (NFBC1966) with accurate national register data, we first report a wide array of perceptions concerning work ability among 46-year-old women with PCOS and non-PCOS controls, accompanied by a prospective 2-year follow-up of registered disability and unemployment days. Finally, we present lifelong follow-up data regarding new disability pensions among women with and without PCOS until 52 years of age.

## Subjects and methods

### Study population

The longitudinal NFBC1966 study started with all expected births in 1966 in the two northernmost provinces of Finland (96% of all births in the region, *n*  = 12 058 live-born babies, 5889 females, all Finnish Caucasian) (14). In the present study, we used data from the 31-year and 46-year follow-ups and from the national registers. Surveys were sent to all cohort members with an address in Finland (5608 women at age 31, response rate 81%; 5123 women at age 46, response rate 72%). The NFBC1966 data were linked with data from Statistics Finland, the Social Insurance Institution of Finland (SII), and the Finnish Center for Pensions (FCP).

**Figure 1:**
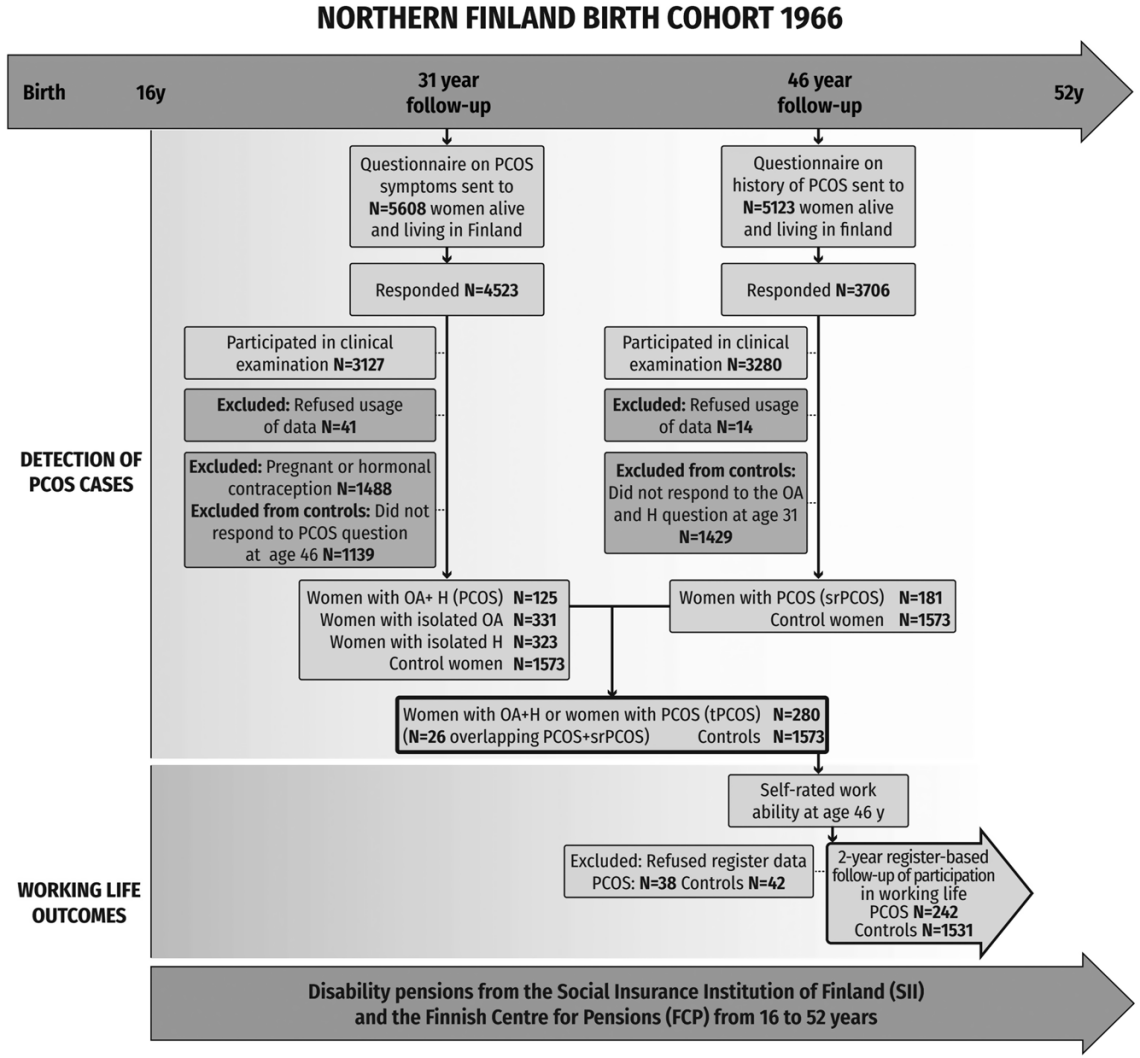
Flowchart of the study design.

As validated earlier (6, 7, 15, 16), women with PCOS were identified from the cohort at two time points: by asking about PCOS symptoms at age 31 and lifetime PCOS diagnosis at age 46. At age 31, after excluding pregnant women and those using hormonal contraceptives, 125 women (4.1%) reported oligomenorrhea and hirsutism and were considered to have PCOS. At age 46, 181 women reported having been diagnosed by a physician with polycystic ovaries (PCOs) and/or PCOS. The total PCOS study group included 280 women (26 women overlapping, i.e. identified in both surveys). The non-PCOS women consisted of all the women who did not report PCOS symptoms at age 31 and who reported not being diagnosed with PCOs/PCOS by age 46 (*n* = 1573). Being identified as a case or non-case of PCOS and responses to work ability questions at age 46 were required to be included in the study. Women who did not grant permission for their data to be used were excluded (*n*_31_ = 41 and *n*_46_ = 14 concerning surveys and *n*  = 80 concerning registered data). The flowchart of the study is presented in Fig. 1.

### Self-rated measures of work ability at age 46

We utilized several items of the Work Ability Index to obtain participants’ perceptions about their work ability (17). First, the ratings on the current Work Ability Score (0–10; where 10 means lifetime best) were classified into good (8–10) vs poor (0–7) work ability (18, 19, 20). Secondly, separate ratings on work ability in relation to physical and mental job demands on a Likert scale (1–5) were dichotomized as good (‘very’ or ‘fairly good’ 4–5) vs poor (1–3). Next, answers (scale 1–6) to the question ‘Do your diagnosed diseases or injuries affect your ability to work?’ were dichotomized as no (‘6. No problems, no diseases’ or ‘5. I can work, with symptoms’) vs yes (I need to change the pace or the way I work: ‘4. Sometimes’ or ‘3. Often’ or ‘2. I should only work part time because of illness’ or ‘1. I am unable to work’). Lastly, we categorized the participants’ own 2-year prospects on ability to work as good (‘fairly sure’) vs poor (‘not sure’ or ‘hardly’).

### Two-year prospective follow-up on registered participation in working life between ages 46 and 48

Individually determined 2-year follow-up periods (730 days) started the day after an individual returned the questionnaire or underwent the clinical examination of the 46-year study. The FCP register covers all days of gainful employment and unemployment. The FCP and SII registers cover most days with disability benefits as detailed below.

The employment status for each day was determined as a disability day, unemployment day, or an employment day based on receiving any type of disability benefit or unemployment compensation, or on being employed or self-employed. In cases of overlaps (e.g. sick leaves on contracted days), ‘disability’ as one of the codes was decisive, and ‘employment’ was coded only with no other coding. As these days were subsidized or paid differentially, we transformed them into 7 days per week.

Disability days cover all medically certified (i.e. disability-related) absences from work, that is sickness absences and disability pensions, either full or part time. Sickness allowances are registered by the SII after varying deductible times, 1+9 weekdays for employees and 1+3 weekdays for entrepreneurs, and in cases of accidents by the FCP after 1+3 weekdays. At most, sickness allowance is paid for 1 year, after which eligibility for a fixed-term or permanent disability pension is evaluated. Unemployment days cover all days subsidized as basic or earnings-based unemployment allowance, labor market subsidy or unemployment training allowance.

### Lifetime emergence of disability pensions until age 52

In Finland, all pensions are registered by either the SII or FCP. Depending on the register, one to three diagnoses for disability are recorded. In our study, the date and the diagnoses for the first-ever granted disability pension of any type for each individual from age 16 to 52 years were determined.

### Confounding factors

All health-related and socioeconomic factors that were considered to potentially influence participation in working life are shown in Table 1.

**Table 1:** Survey-based characteristics and register-based employment status at age 46 (numbers of cases and proportions) among the study population.

	Non-PCOS controls		PCOS		*P*-value
	*n*	%	*n*	%	
Self-rated health	1563		240		<0.001
Good	1070	68.5	133	55.4	
Poor	493	31.5	107	44.6	
BMI	1544		238		<0.001
<25	775	50.2	92	38.7	
25–30	470	30.4	74	31.1	
>30	299	19.4	72	30.3	
Alcohol consumption	1564		241		0.396
Abstinence	193	12.3	37	15.4	
Low-risk drinking	1248	79.8	184	76.3	
High-risk drinking	123	7.9	20	8.3	
Smoking	1557		236		0.433
No smoking	886	56.9	130	55.1	
Previous/occasional smoking	345	22.2	49	20.8	
Regular smoking	326	20.9	57	24.2	
Physical activity	1565		241		0.405
Low	337	21.5	55	22.8	
Moderate	645	41.2	107	44.4	
High	583	37.3	79	32.8	
Parity	1449		226		0.797
0	142	9.8	22	9.7	
1–2	783	54.0	120	53.1	
>3	524	36.2	84	37.2	
Residential area	1554		244		0.015
Urban	1028	66.2	142	58.2	
Rural	526	33.8	102	41.8	
Marital status	1573		280		0.027
Single	351	22.3	46	16.4	
In a relationship	1222	77.7	234	83.6	
Education	1571		241		0.189
Basic	86	5.5	19	7.9	
Secondary	997	63.5	157	65.1	
Tertiary	488	31.1	65	27.0	
Employment history	1508		235		0.419
Continuous	1047	69.4	157	66.8	
Discontinuous	461	30.6	78	33.2	
Employment status at age 46	1493		238		<0.001
Employment day	1308	87.6	187	78.6	
Unemployment day	102	6.8	28	11.8	
Disability day	83	5.6	23	9.7	

At age 46, self-rated health was assessed by asking ‘How would you estimate your current health status?’ and categorized as good (very good, good) vs poor (moderate, poor, very poor). Weight and height were measured with a digital scale and a standard calibrated stadiometer. If measurements were missing, self-reported values were used. BMI (kg/m^2^) was categorized into three groups: normal (<25 kg/m^2^), pre-obese (25–30 kg/m^2^), and obese (>30 kg/m^2^). Alcohol consumption (types of beverages, frequencies, and quantities) was categorized into three groups: abstinence, low-risk drinking (≤20 g/day), or high-risk drinking (>20 g/day) (21, 22). Smoking status was trichotomized into never smoking, previous/occasional smoking, and regular smoking. Leisure-time physical activity (types and frequencies of activities) was categorized into low, moderate, and high (23). Parity was categorized into no, one to two, and three or more deliveries.

Self-reported residential area was dichotomized into urban vs rural. Marital status was dichotomized into single vs in a relationship. Primarily based on register data of Statistics Finland and secondarily from the survey, the lifetime highest level of education was classified as basic (9 years), secondary (matriculation examination or vocational education), or tertiary (short-cycle tertiary or university level) education. Self-rated employment history was dichotomized into continuous (always or mostly working on permanent or long work contracts) or discontinuous (long or short work contracts with occasional unemployment periods, mainly short contracts, mostly unemployed, mostly supported working, or never in paid employment).

In addition, the registered employment status on the first day of the 2-year follow-up (employment, disability, or unemployment day) starting from the 46-year study was recorded.

### Statistical analysis

Statistical analyses were conducted using IBM SPSS Statistics 25 and R 3.6.1. We assessed the associations of PCOS with the categorical potential confounders and the dichotomized work ability measures using cross-tabulations and the χ^2^ and likelihood ratio tests. We evaluated the differences in the accumulation of disability and unemployment days during the 2-year follow-up periods among the women with and without PCOS and according to the categories of potential confounders using the Mann–Whitney *U* test in dichotomized factors and Kruskal–Wallis tests in trichotomized factors. A two-sided *P*-value <0.05 was considered statistically significant.

The associations between self-rated measures of poor work ability and PCOS status were further analyzed with binary logistic regression models and reported as odds ratios (ORs) with 95% CIs, first unadjusted. Prior to building the different models (1–3), we ran univariate analyses. Thereafter, we tested the models in multivariate analysis; (i) health-related variables: self-rated health, BMI, alcohol consumption, smoking, physical activity, and parity; (ii) socioeconomic variables: residential area, marital status, level of education, and employment history; and (iii) employment status at age 46.

We used Poisson regression analyses to calculate the 2-year incidence rate ratios (IRRs) and their 95% CIs for disability and unemployment days (separate models for each) for the women with PCOS and non-PCOS controls, with analogs adjustments.

Finally, we calculated median ages with interquartile ranges (IQR) at first disability pension among women with PCOS and non-PCOS controls and used Kaplan–Meier analysis (Mantel-Cox estimate) to estimate hazard ratios (HRs) with 95% CIs for the lifetime emergence of disability pension in the study groups until 2018, when the participants turned 52 years of age.

### Ethical permissions and written consent

The ethics committee of Northern Ostrobothnia hospital district approved the study (EETTMK:94/2011). All participants provided informed consent for the use of their data as well as linkages to national registers (24, 25). The NFBC1966 study was conducted in accordance with the Helsinki Declaration.

## Results

At age 46, the women with PCOS evaluated their health as poor more often (44.6% vs 31.5%, *P* < 0.001) and were more often obese than non-PCOS women (30.3% vs 19.4%, *P* < 0.001) (Fig. 1 and Table 1). Regarding socioeconomic factors, women with PCOS were more often in a relationship (83.6% vs 77.7%, *P* = 0.027) and rural residents than the referents (41.8% vs 33.8%, *P* = 0.015). Based on register data at age 46, women with PCOS were more often on disability leave (9.7% vs 5.6%, *P* < 0.001) or unemployed (11.8% vs 6.8%, *P* < 0.001) compared to their non-PCOS counterparts (Table 1).

Concerning self-ratings, the women with PCOS more often reported poor work ability in general (23.7% vs 18.1%, *P* = 0.039) from the perspective of physical job demands (8.9% vs 5.0%, *P* = 0.016) but not in terms of mental job demands. Of note, women with PCOS more often reported having poor work ability due to diseases (26.6% vs 19.7%, *P* = 0.015) and were less likely expecting their health would allow working in their current profession after 2 years compared to non-PCOS women (14.5% vs 9.7%, *P* = 0.025). When these associations were tested in multivariate logistic regression analyses, the higher odds for any self-rated poor work ability measure were diluted to non-significance when adjusted for self-rated health (Table 2). Regarding other adjustments, in PCOS women, the higher risks for poor work ability in relation to physical job demands (OR: 1.85; 95% CI: 1.12–3.06), in relation to diseases (OR: 1.45; 95% CI: 1.08–2.01), regarding their own 2-year-prospects (OR: 1.58; 95% CI: 1.06–2.36) were largely retained despite adjustments for health behaviors or socioeconomic factors.

**Table 2:** Odds ratios (ORs) and their 95% CIs for self-rated poor work ability at age 46 in women with PCOS in comparison to non-PCOS controls (OR = 1). Statistically significant values (*P* < 0.05) are presented in bold.

	Poor work ability score ≤7	Poor work ability in relation to physical job demands	Poor work ability in relation to mental job demands	Perceived disability at work because of diseases	Poor self-prognosis of work ability 2 years from now
Unadjusted OR (95% CI)	**1.41 (1.02–1.96)**	**1.85 (1.12–3.06)**	1.21 (0.62**–**2.33)	**1.47 (1.08–2.01)**	**1.58 (1.06–2.36)**
Health-related adjustments					
Self-rated health	1.09 (0.76–1.57)	1.39 (0.82–2.36)	0.91 (0.46–1.78)	1.25 (0.89–1.75)	1.27 (0.83–1.95)
BMI	1.31 (0.93–1.82)	**1.67 (1.00–2.79)**	1.05 (0.53–2.10)	**1.40 (1.02–1.93)**	1.48 (0.98–2.23)
Alcohol consumption	**1.39 (1.00–1.94)**	**1.80 (1.08–3.00)**	1.20 (0.62–2.34)	**1.45 (1.06–1.99)**	**1.56 (1.04–2.34)**
Smoking	1.39 (0.99–1.94)	**1.81 (1.09–3.02)**	1.07 (0.54–2.13)	**1.50 (1.10–2.07)**	**1.57 (1.04–2.36)**
Physical activity	**1.40 (1.00–1.95)**	**1.84 (1.10–3.07)**	1.23 (0.63–2.39)	**1.46 (1.06–2.00)**	**1.55 (1.03–2.32)**
Parity	1.37 (0.97–1.93)	1.69 (0.99–2.91)	1.11 (0.54–2.28)	**1.48 (1.06–2.05)**	**1.55 (1.01–2.37)**
Socioeconomic adjustments					
Residential area	1.39 (1.00–1.94)	**1.94 (1.17–3.23)**	1.32 (0.68–2.57)	**1.47 (1.07–2.02)**	**1.56 (1.04–2.35)**
Marital status	**1.43 (1.03–1.99)**	**1.90 (1.14–3.15)**	1.22 (0.63–2.36)	**1.49 (1.08–2.04)**	**1.59 (1.06–2.38)**
Education	1.37 (0.98–3.08)	**1.76 (1.06–2.93)**	1.16 (0.60–2.25)	**1.44 (1.05–1.97)**	**1.52 (1.01–2.29)**
Employment history	1.39 (0.99–1.94)	**1.79 (1.06–3.02)**	1.15 (0.58–2.29)	**1.45 (1.05–1.99)**	**1.54 (1.02–2.32)**
^a^Model 1	1.06 (0.72–1.57)	1.25 (0.70–2.25)	0.69 (0.30–1.61)	1.29 (0.91–1.84)	1.26 (0.79–2.01)
^b^Model 2	1.36 (0.97–1.91)	**1.88 (1.11–3.19)**	1.24 (0.62–2.49)	**1.43 (1.04–1.98)**	1.50 (0.98–2.28)
^c^Model 3	1.02 (0.69–1.52)	1.26 (0.70–2.29)	0.73 (0.31–1.70)	1.26 (0.88–1.81)	1.20 (0.74–1.95)

^a^Model 1: health-related adjustments: BMI, self-rated health, alcohol consumption, smoking, physical activity, parity; ^b^Model 2: socioeconomic adjustments: residential area, education, marital status, employment history; ^c^Model 3: All: BMI, self-rated health, alcohol consumption, smoking, physical activity, parity, residential area, education, marital status, employment history.

During the 2-year follow-up starting from the 46-year survey, women with PCOS had on average 32 additional days of registered disability (mean 72 vs 40, *P* = 0.008) and 31 additional days of unemployment (mean 90 vs 59, *P* = 0.003) compared to the non-PCOS referents (Supplementary Table 1, see section on supplementary materials given at the end of this article). Correspondingly, women with PCOS had a higher risk for disability days (IRR: 1.77; 95% CI: 1.74–1.80) and unemployment days (IRR: 1.52; 95% CI: 1.49–1.54) compared to non-PCOS women in the unadjusted Poisson regression analyses (Table 3). The risks for disability and unemployment days remained significantly elevated after considering grouped health-related factors (IRR: 1.29; 95% CI: 1.26–1.31 and IRR: 1.29; 95% CI: 1.27–1.31, respectively) and socioeconomic factors (IRR: 1.63; 95% CI: 1.60–1.66 and IRR: 1.44; 95% CI: 1.41–1.46), and even in the conservative analysis adjusting for the baseline employment status of the prospective follow-up (IRR: 1.19; 95% CI: 1.17–1.21 and IRR: 1.09; 95% CI: 1.07–1.11), as well as when considering all of the aforementioned covariates (IRR: 1.25; 95% CI: 1.22–1.27 and IRR: 1.26; 95% CI: 1.23–1.28). The strongest single factors affecting disability and unemployment risk were registered employment status and self-rated health status at the follow-up baseline (Table 3).

**Table 3:** Incidence rate ratios (IRRs) and their 95% CIs of registered disability days and unemployment days in women with PCOS during the 2-year follow-up period (730 days) starting from the 46-year survey. Among non-PCOS controls, IRR = 1.

	Disability days	Unemployment days
Unadjusted	1.77 (1.74–1.80)	1.52 (1.49–1.54)
Health-related adjustments		
Self-rated health	1.38 (1.36–1.41)	1.40 (1.38–1.42)
BMI	1.59 (1.56–1.62)	1.42 (1.40–1.44)
Alcohol consumption	1.67 (1.64–1.70)	1.52 (1.50–1.54)
Smoking	1.70 (1.67–1.73)	1.43 (1.41–1.45)
Physical activity	1.75 (1.72–1.78)	1.52 (1.50–1.54)
Parity	1.64 (1.61–1.67)	1.50 (1.47–1.52)
Socioeconomic adjustments		
Residential area	1.78 (1.75–1.81)	1.49 (1.47–1.51)
Marital status	1.80 (1.77–1.83)	1.55 (1.52–1.57)
Education	1.64 (1.61–1.67)	1.46 (1.44–1.49)
Employment history	1.66 (1.63–1.69)	1.44 (1.42–1.47)
Employment status at age 46	1.19 (1.17–1.21)	1.09 (1.07–1.11)
^a^Model 1	1.29 (1.26–1.31)	1.29 (1.27–1.31)
^b^Model 2	1.63 (1.60–1.66)	1.44 (1.41–1.46)
^c^Model 3	1.19 (1.17–1.21)	1.09 (1.07–1.11)
^d^Model 4	1.25 (1.22–1.27)	1.26 (1.23–1.28)

^a^Model 1: health-related adjustments: BMI, self-rated health, alcohol consumption, smoking, physical activity, parity; ^b^Model 2: socioeconomic adjustments: residential area, education, marital status, employment history; ^c^Model 3: employment status at age 46; ^d^Model 4: all: BMI, self-rated health, alcohol consumption, smoking, physical activity, parity, day 1 status, residential area, education, marital status, employment history.

**Figure 2:**
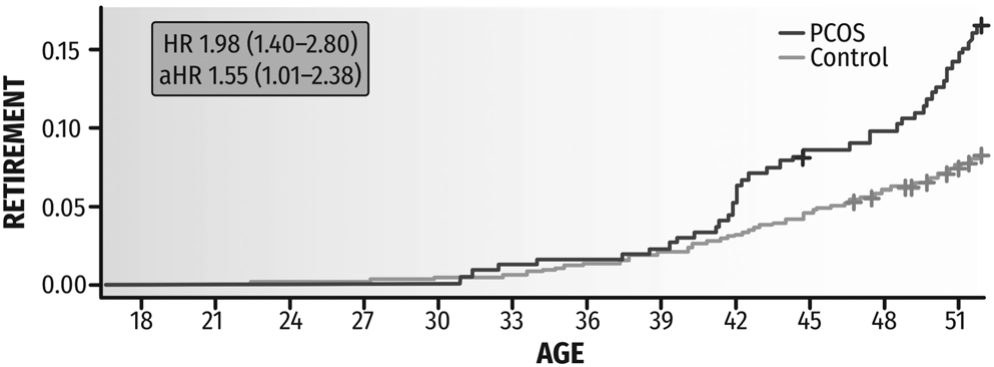
Cumulative hazard functions of age at first-ever disability pension decision in women with PCOS and in non-PCOS controls. Women with PCOS: *n*  = 43, 15.4% vs non-PCOS control women: *n*  = 126, 8.0%, *P*-value <0.001. The symbols ‘+’ indicate individuals censored from follow-up due to death, and the final time point of follow-up at age 52 years. The figure includes the unadjusted hazard ratio (HR) and the fully adjusted HR (aHR, adjusted for self-rated health, BMI, alcohol consumption, smoking, physical activity, parity, marital status, type of residential area, education, employment history) with their 95% CIs from Kaplan–Meier and Cox regression analyses for disability retirement.

Concerning lifetime disability retirement by age 52, women with PCOS had been granted a disability pension more often than women without PCOS (*n* = 43, 15.4% vs *n* = 126, 8.0%, *P* < 0.001) and at younger age (median (IQR) 44.6 (41.5–50.3) vs 44.9 (39.4–49.9)). In the unadjusted Kaplan–Meier analysis, the lifetime risk for such pre-term retirement was two-fold greater in women with PCOS (HR: 1.98; 95% CI: 1.40–2.80) and 1.6-fold greater in the Cox model adjusting for all potential confounders (HR: 1.55; 95% CI: 1.01–2.38) as compared to non-PCOS women (Fig. 2). Of all diagnoses recorded (one to three per decision) for any disability pension decision, the most common was from the ICD-10 class F (mental and behavioral disorders), which were included in 46.5 and 46.0% of decisions for women with and without PCOS, respectively. The corresponding figures for class M (diseases of the musculoskeletal system and connective tissue) were 18.6 and 27.0%, respectively. There was no statistical difference concerning diagnoses (*P* = 0.450). Notably, no ICD-10 diagnoses from class N (diseases of the genitourinary system) or E28.2 (PCOS) were recorded.

## Discussion

In this first general population-based study, we reported lower work ability in middle-aged women with PCOS based on both self-rated outcomes and prospective register-based follow-ups and life course follow-ups of disability retirements, considering several potential confounders. At age 46, women with PCOS reported several dimensions of poorer work ability, especially in relation to physical job demands, and these associations seemed to be linked with poorer general health. During the consequent register-based follow-up, the women with PCOS had on average an additional month of disability-based absences and unemployment days compared to non-PCOS women in only 2 years, corresponding to 25% higher incidence rates in the multivariate analyses. Finally, the HR for disability retirement by age 52 was doubled for women with PCOS compared to non-PCOS women, with a 1.6-fold greater risk after adjustments.

To our knowledge, only one study has evaluated retirement among women with PCOS, as one dimension of socioeconomic status. This large Danish population study (13) had a cross-sectional, clinically rooted case–control design and reported that women with PCOS retired more often than non-PCOS women (*n* = 870, 6% vs *n* = 1529, 4%, respectively). Our results, presenting cumulative risks in a total population sample, extend this evidence by showing that women with PCOS have an increased risk for early disability retirement compared to women without the condition (15.4% vs 8%, respectively). Alarmingly, as depicted for the first time by our Kaplan–Meier analysis, the retirement curves of women with and without PCOS started to deviate as early as 40 years of age.

Our results are best interpreted in light of the large-scale comorbidities associated with PCOS. In the same cohort, we have previously reported PCOS as carrying a risk for multimorbidity regarding infertility, obesity, dyslipidemia, high blood pressure, cardiovascular events, anxiety, depression, and psychosis (5, 6, 7, 10, 15, 26). Likewise, other studies have reported large-scale comorbidities in PCOS, such as metabolic derangements, asthma, migraine, musculoskeletal disorders, and mental distress (3, 4). Importantly, the accumulation of comorbidities during the life course seems to start earlier among women with PCOS than among non-PCOS women (4). It is well established that over 50% of women with PCOS are obese (6, 27), and we were the first to show that this BMI deviation starts already before the age of 5 years, thus posing long-term health risks and multimorbidity (7). Notably, clustered health risks and consequent multimorbidity, defined as the co-occurrence of two or more chronic conditions, typically reduce functional reserve, with an elevated risk of losing the ability to work (28).

Accordingly, in this study, the major factor shaping the association between poor work ability and PCOS was self-rated general health, a widely used global measure of health, disability, and mortality, also known to effectively gauge multimorbidity in epidemiological studies (12, 28, 29, 30). Self-rated general health serves as a reliable measure of multimorbidity in younger populations not yet largely impacted by major age-related diseases (10, 31). Indeed, in our study, women with PCOS reported poorer work ability regarding their diseases and physical job demands, regardless of socioeconomic factors. Interestingly, even though the increased rate of mental distress in PCOS is also well established in this study set (5, 10), the affected women did not report poor work ability regarding mental job demands. We note that the study groups did not differ by educational level, which largely reflects the type of job demands. Lastly, although obesity has been shown to associate with poorer work ability in our cohort (20) and suggested to play a role in the impact of PCOS on occupational status (13), adjusting for obesity or risky health behaviors did not dilute the association of PCOS with poorer work ability, disability, or unemployment days in the present study. In sum, not mere risk factors but more severe actual health conditions gauged by an impaired self-rated general health status appeared to generate negative subjective and registered outcomes of work disability in association with PCOS.

The results of our 2-year follow-up of registered unemployment raise concerns about employability among women with PCOS. Regrettably, people with poor health are not necessarily granted disability-based compensation in cases of poor work ability. Instead, with inadequate multidisciplinary care and rehabilitation, they may drift into unemployment (12, 32, 33).

Methodologically, the NFBC1966 provides an excellent platform for screening associations between health and working life. Because the participants were born around the same year, any risk of bias arising from macroeconomic fluctuations affecting working life circumstances and employment rates was low. In comparison to any study conducted among highly selected PCOS populations from infertility clinics, our unselected general population data represent subjects from all sectors of the economy and occupations. The PCOS case detection strategy has been well established and described in our previous publications (5, 6, 16) and supported by other researchers (34). Regarding generalizability, as our cohort members were ethnically Finnish Caucasians living in a Nordic welfare state that provides access to education and healthcare to all citizens, further studies are needed in other countries, among other ethnicities and with different age groups. Nevertheless, we used internationally validated survey questions related to work ability and could consider a wide set of established potential confounders. Importantly, by using highly reliable and accurate register data on actual retirement, disability days, and unemployment days, we avoided recall biases. As a limitation of this study, the registers do not fully cover short sickness absences, whereas those exceeding 2 weeks are comprehensively registered. Therefore, our measurements of disability days are underestimates. Lastly, as in any observational study, unmeasured, uncontrolled confounders always remain a possibility.

The prevalence of PCOS is rising internationally. In 2017, the global age-standardized incidence rate was estimated as 82 per 100 000 women of reproductive age, accompanied by 22 disability-adjusted life years (35). Accordingly, PCOS causes an enormous economic burden for society. For example, in the United States, PCOS has been estimated to cost $4.36 billion annually, covering only healthcare costs related to initial evaluation, treatment of menstrual dysfunction, infertility, diabetes, and hirsutism (36). The high rates of PCOS-related disability and unemployment days and retirement found in this study represent significant productivity losses for individuals, employers, and societies, warranting future studies on the comprehensive costs of this major public health condition.

For healthcare practitioners, recognizing the relevance of PCOS in relation to working life outcomes is imperative. In fact, we suggest considering PCOS as a red flag for detecting women at high risk for multimorbidity and consequent disability. As for clinical feasibility, only two targeted questions on menstrual irregularity and excessive hair growth, or paying attention to PCOS diagnosis, seem useful for identifying a female population at high risk for poor working life outcomes. Actions following this risk recognition, including consideration of timely care and rehabilitation for the co-morbidities, should be developed to prevent permanent disability. Promisingly, recent developments in enabling participation in working life among workers with chronic illnesses provide further possibilities for supporting work ability in workplace practices and in occupational health services (37).

We conclude that having PCOS predicts poorer participation in working life by increasing the risk of disability-based absences and unemployment and disability retirement in middle age. As such, concerted efforts should be made to support sustainable working careers for women with PCOS.

## Supplementary Material

Supplementary Table 1. Registered two-year follow-up of participation in working life starting from the 46-year study. Mean and median counts of registered disability and unemployment days during the individually determined two-year follow-up periods (730 days) in women with and without PCOS, and in relation to all covariates at age 46. With highly skewed distributions of the days, the medians are only marked if not zero.

## Declaration of interest

Terhi Piltonen is on the editorial board of *European Journal of Endocrinology*. Terhi Piltonen was not involved in the review or editorial process for this paper, on which they are listed as an author. The other authors declare that there is no conflict of interest.

## Funding

This study was supported by grants from the Academy of Finland http://dx.doi.org/10.13039/501100002341 (grant numbers 315921, 321763), The Finnish Cultural Foundation (grant number 60212369), The Päivikki and Sakari Sohlberg Foundation (grant number 6207), The Emil Aaltonen Foundation (grant number 210083K). NFBC1966 received financial support from University of Oulu Grant no. 24000692, Oulu University Hospital Grant no. 24301140, and ERDF European Regional Development Fund http://dx.doi.org/10.13039/501100008530 Grant no. 539/2010 A31592.

## Author contribution statement

L K, R K A, T T P, and L A-M conceived the research question and drafted the study design. E V acquired the data for the registered variables. L K and E V performed the statistical analyses. L K wrote the first draft of the manuscript. All authors contributed to the interpretation of the results, critically revised the manuscript, and approved the final version before submission. T T P and L A-M are the guarantors of the study. L A-M and T T P contributed equally to this work.
