# Supplementary material for: Women with polycystic ovary syndrome have poorer work ability and higher disability retirement rate at midlife: a Northern Finland Birth Cohort 1966 study
Source: Eur J Endocrinol. 2022 Jul 13;187(3):479–88. doi: 10.1530/EJE-22-0027 (PMC9422246; doi:10.1530/EJE-22-0027)
Supplement: Supplementary Table 1. Registered two-year follow-up of participation in working life starting from the 46-year study. Mean and median counts of registered disability and unemployment days during the individually determined two-year follow-up periods (730 days) in women with and without PCOS, and in [file supplementary_table_1.pdf]

Supplementary data

Supplementary Table 1. Registered two-year follow-up of participation in working life starting from the 46-year study. Mean and median counts of registered disability and unemployment days during the individually determined two-year follow-up periods (730 days) in women with and without PCOS, and in relation to all covariates at age 46. With highly skewed distributions of the days, the medians are only marked if not zero.

	**N**		**Disability days (range 0–730) Mean (Median if not=0)**			**Unemployment days (range 0–730) Mean (Median if not=0)**		
	**Non-PCOS**	**PCOS**	**Non-PCOS**	**PCOS**	*p*	**Non-PCOS**	**PCOS**	*p*
**All participants**	1531	242	40	72	***0.008***	59	90	***0.003***
**In relation to covariates at age 46:**								
**Self-rated health *(p)***			***<0.001***	***0.005***		***<0.001***	*0.132*	
Good	1043	132	15	32		44	67	
Poor	478	105	96	117		90	119	
**BMI**			***<0.001***	*0.254*		*0.431*	*0.361*	
<25	755	91	37	65		49	72	
25–30	460	74	30	58		62	86	
>30	288	70	64	80		78	110	
**Alcohol consumption**			***0.041***	*0.647*		***0.028***	*0.685*	
Abstinence	181	37	86	81		68	119	
Low-risk drinking	1223	182	32	70		52	85	
High-risk drinking	119	19	51	45		107	80	
**Smoking**			***<0.001***	*0.135*		***0.001***	*0.742*	
No smoking	857	128	28	40		46	103	
Previous/occasional	343	49	47	113		56	65	
Regular smoking	315	56	70	104		97	67	
**Physical activity**			***0.001***	*0.496*		***<0.001***	***0.014***	
Low	325	53	64	103		83	104	
Moderate	627	107	39	74		65	119	
High	571	78	25	41		39	40	
**Parity**			***0.027***	***0.034***		*0.813*	*0.341*	
0	142	22	53	39		62	183	
1–2	759	119	32	42		58	83	
>3	515	82	49	109		57	67	
**Residential area**			*0.633*	*0.308*		***0.029***	*0.531*	
Urban	1013	141	41	59		55	86	
Rural	516	101	39	80		68	94	
**Marital status**			*0.734*	*0.397*		***<0.001***	*0.601*	
Single	334	44	51	110		88	94	
In a relationship	1197	198	37	63		51	86	
**Education**			***<0.001***	*0.266*		***<0.001***	*0.267*	
Basic	81	18	103	171		122	130	
Secondary	970	155	42	78		65	98	
Tertiary	478	65	28	21		36	58	
**Employment history**			***0.006***	*0.669*		***<0.001***	***<0.001***	
Continuous	1029	156	27	46		22	55	
Discontinuous	440	76	63	101		144	158	
**Self-rated work ability**			***<0.001***	***<0.001***		***<0.001***	*0.145*	
Good	1209	179	12	20		48	68	
Poor	262	54	166	223 (20)		103	156	
**Registered employment status at age 46**			***<0.001***	***<0.001***		***<0.001***	***<0.001***	
Employment day	1308	187	20	13		24	33	
Unemployment day	102	28	32	22		482 (545)	477 (498)	
Disability day	83	23	494 (730)	561 (730)		31	29	

7
